# Supported Pd-Au Membrane Reactor for Hydrogen Production: Membrane Preparation, Characterization and Testing

**DOI:** 10.3390/molecules21050581

**Published:** 2016-05-09

**Authors:** Adolfo Iulianelli, Marjan Alavi, Giuseppe Bagnato, Simona Liguori, Jennifer Wilcox, Mohammad Reza Rahimpour, Reza Eslamlouyan, Bryce Anzelmo, Angelo Basile

**Affiliations:** 1 Institute on Membrane Technology of the Italian National Research Council (TM-CNR), Cubo 17/C, University of Calabria, Rende 87036, Italy; marjan.alavi@ymail.com (M.A.); gb_87@hotmail.it (G.B.); 2 Department of Chemical Engineering, School of Chemical and Petroleum Engineering, Shiraz University, Shiraz 71345, Iran; rahimpor@shirazu.ac.ir (M.R.R.); eslamlo@shirazu.ac.ir (R.E.); 3 Department of Energy Resources Engineering, Stanford University, Stanford 94305, CA, USA; sliguori@stanford.edu (S.L.); wilcoxj@stanford.edu (J.W.); brlz@stanford.edu (B.A.)

**Keywords:** Pd-Au membrane, methane steam reforming, composite membrane, hydrogen production

## Abstract

A supported Pd-Au (Au 7wt%) membrane was produced by electroless plating deposition. Permeation tests were performed with pure gas (H_2_, H_2_, N_2_, CO_2_, CH_4_) for long time operation. After around 400 h under testing, the composite Pd-Au membrane achieved steady state condition, with an H_2_/N_2_ ideal selectivity of around 500 at 420 °C and 50 kPa as transmembrane pressure, remaining stable up to 1100 h under operation. Afterwards, the membrane was allocated in a membrane reactor module for methane steam reforming reaction tests. As a preliminary application, at 420 °C, 300 kPa of reaction pressure, space velocity of 4100 h^−1^, 40% methane conversion and 35% hydrogen recovery were reached using a commercial Ni/Al_2_O_3_ catalyst. Unfortunately, a severe coke deposition affected irreversibly the composite membrane, determining the loss of the hydrogen permeation characteristics of the supported Pd-Au membrane.

## 1. Introduction

The current development in energy use is oriented towards reducing carbon consumption due to its environmental pollution. Hydrogen as a clean and sustainable energy carrier has gained more attention during the past decades. When hydrogen reacts with oxygen in fuel cells and internal combustion engines, a large amount of energy is released explosively in heat engines and quietly in fuel, releasing water as the product.

The present source of hydrogen comes mainly from synthesis gas, which is a mixture of H_2_, CO and CO_2_, and it is produced by breaking the strong C-H bonds (439 kJ/mol) of hydrocarbons in reforming reactions. Afterwards, hydrogen is purified and separated by different energy intensive steps. A membrane reactor (MR) technology can represent an energetically efficient option to the conventional processes, with the practical advantages of a smaller footprint and capital cost reduction. In this alternative reformer, the hydrogen is produced and continuously removed from the reaction side for permeation through a hydrogen perm-selective membrane, shifting the reaction towards further product formation. As a consequence, the conversion increases and pure hydrogen is produced at the same time. 

In the specialized literature, porous carbon, silica and zeolite membranes have been used for hydrogen separation [1,2,3]. Nevertheless, their separation characteristics are still not acceptable although their cost is relatively low. Dense membranes made of palladium (Pd) are able to separate hydrogen from a gas mixture by a solution-diffusion mechanism with a theoretically infinite hydrogen perm-selectivity [4]. As it is well known, the cost of Pd-membranes is relatively high, but it can be reduced by depositing a thin Pd layer on a metallic or ceramic support [5]. 

Despite of the great permeation characteristics of Pd, its application is restricted by some factors. Hydrogen embrittlement of Pd-membranes occurs when the operating conditions are below 300 °C and 2.0 MPa. In these conditions, hydrogen permeation through the membrane allows for the change of Pd lattice from α to β phase, and *vice versa*. Several cycles from α to β phase can cause the formation of cracks in the membrane lattice [6].

Furthermore, dense Pd-membranes are affected by surface contamination. Different studies have demonstrated the effects of different contaminants such as CO [7,8,9,10], H_2_O [11,12,13], Cl [14], NH_3_ [15], certain hydrocarbons and sulfur compounds like H_2_S [16] on the membranes. In particular, by exposing H_2_S to the Pd surface, sulfur is reversibly adsorbed on the palladium surface blocking the adsorption sites for hydrogen with a reduction in the hydrogen permeation. Sulfur can also react with the Pd surface producing Pd_4_S which acts as a barrier to hydrogen permeation decreasing the permeance [17]. The lattice constants of Pd and Pd_4_S are highly different, so this can cause cracks in the membrane [18].

In order to overcome these drawbacks, some studies proposed to change the crystal structure into nanostructure [19]. However, most of the researches proposed an enhancement of the membranes stability by alloying them with other elements [20,21,22]. Most efforts in this area are dedicated to Pd-Cu alloys because of their high resistance to sulfur poisoning [23,24,25]. Lately, the Pd-Au membranes have been studied due to their higher hydrogen permeability with respect to Pd-Cu. In the pioneeristic study realized by McKinely *et al.* [26,27], it was shown that the addition of Au content in the range 0wt% –20wt% to palladium membranes enhances the tolerance to sulfur compounds, minimizing the embrittlement phenomenon and improving the hydrogen permeability more than pure Pd. In the experiments done by Way *et al.* [28], the Pd-15%wt Au has exhibited a higher hydrogen permeating flux in the presence of H_2_S than a Pd-6%wt Cu in the absence of H_2_S. In particular, by using Pd-15%wt Au the hydrogen flux decreased 38% after the exposure to 5 ppm H_2_S at 400 °C, while, with Pd-6%wt Cu, the hydrogen flux was 71% lower than that measured with Pd-Au in H_2_S presence. Chen and Ma [29] examined a Pd-8%wt Au on a porous metal support. In the temperature range between 350 and 500 °C, no sulfide was detected in the membrane even at 54.8 ppm H_2_S exposure.

Some studies have compared hydrogen permeation in Pd-Au alloys with pure Pd. Sonwane *et al.* [30] predicted that the hydrogen permeability in Pd-Au membrane increased by increasing the Au content, having a maximum value at 12wt% Au and showing a hydrogen permeability 1.8 times greater than pure Pd at 180 °C. In other investigations, Gryaznov [31] found that the hydrogen permeability of Pd-10%wt Au at 500 °C is 2.2 times higher than that of pure Pd. Ma *et al.* [32] investigated the performance of several Pd-Au membranes with different Au content at (4.2wt%–16.7wt%) from 250 to 450 °C, confirming that the membranes with 4.2wt% and 5.4wt% Au show higher hydrogen permeability than pure Pd.

However, the stability of Pd-Au membranes under long-term operation has been tested only in a few studies, including the experimental efforts of Guazzone *et al.* and Mardilovich *et al.*, who analyzed the long-term permeation test results of Pd and Pd-Au composite membranes under desulfurized coal-derived syngas at pilot scale [33,34,35]. 

The objective of the present work is to investigate the long-term stability characteristics of hydrogen permeation of a composite membrane constituted of a Pd-Au (Au 7wt%) dense layer supported on a porous stainless steel (PSS) support, meanwhile evaluating the H_2_/N_2_, H_2_/CO_2,_ H_2_/CH_4_ and H_2_/He ideal selectivities. 

Furthermore, at stable value of hydrogen permeance, the composite Pd-Au membrane was allocated in a MR module for carrying out methane steam reforming (MSR) reaction for producing hydrogen and to analyze the effect of coke formation during the course of experiments.

## 2. Results and Discussion

### 2.1. Permeation Tests on the Pd-Au/PSS Membrane

The permeation tests on the supported Pd-Au membrane at the onset of the experimental testing were carried out with pure H_2_ and N_2_ at 350 °C and transmembrane pressures of 50 and 100 kPa, with the results resumed in Table 1.

**Figure 1:**
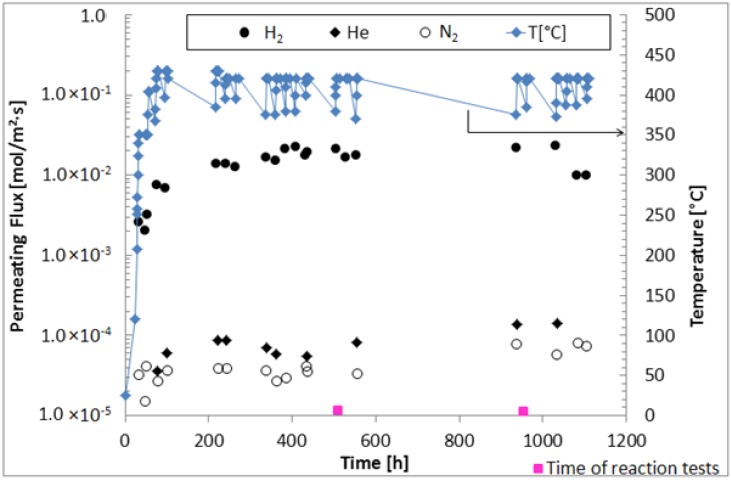
Pure H_2_, N_2_, He permeating fluxes through the Pd-Au/PSS membrane and operating temperature *vs.* time.

As expected, a higher transmembrane pressure affects positively the hydrogen permeation driving force (in this case, represented by the hydrogen partial pressure difference between retentate and permeate sides), making higher hydrogen permeating flux achievable. Nevertheless, the results do not show high performance of the membrane in terms of H_2_/N_2_ ideal selectivity, which can be the consequence of defects, non-homogeneity of Pd-Au layer or Pd-Au alloy not yet formed. Furthermore, it is worth of noting that, at the beginning of the experimental campaign (Figure 1), the composite membrane was probably not at steady state conditions. This should justify why the H_2_/N_2_ ideal selectivity trend increases slightly by increasing the transmembrane pressure from Δp = 50 kPa to Δp = 100 kPa.

**Figure 2:**
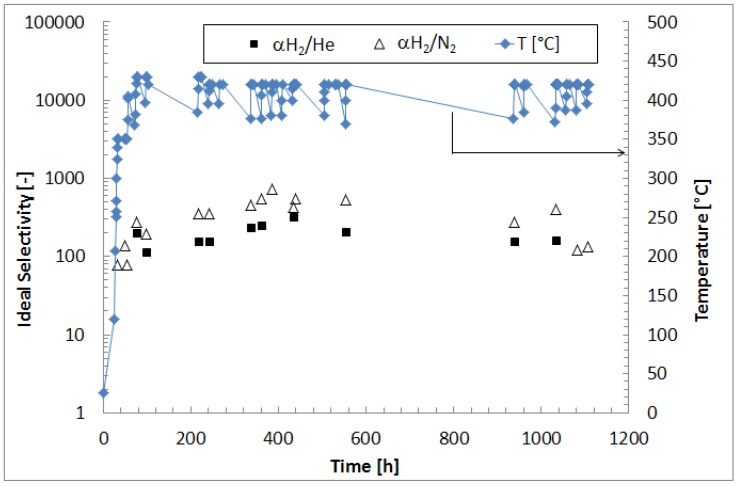
H_2_/N_2_, H_2_/He ideal selectivities for the Pd-Au/PSS membrane and operating temperature *vs.* time.

Then, in order to evaluate the permeation characteristic of the membrane at steady state conditions, further permeation tests with pure N_2_, He and H_2_ were successively performed and, after each cycle of pure gas permeation test, the composite membrane was flowed under hydrogen at Δp = 50 kPa also over night. The results of these tests in terms of H_2_, N_2_ and He permeating fluxes are also resumed in Figure 1. As shown, in the range 0–100 h, the H_2_ permeating flux increased from ~2.0 × 10^−3^ mol/m^2^·s to 7.5 × 10^−3^ mol/m^2^·s, due to the temperature increase from 300 °C to 420 °C. Afterwards, at the set temperature (420 °C) and with the composite membrane exposed to pure hydrogen permeation (a part from the daily realization of pure gas permeation tests), after 400 h, the hydrogen permeating flux reached the steady state condition with an average value of ~2.0 × 10^−2^ mol/m^2^·s, remaining constant up to 1100 h. This trend confirms the optimum annealing conditions for the formation of Pd-Au alloy over this time [32]. As shown in Figure 2, H_2_/N_2_ and H_2_/He ideal selectivities increased in the range 0–100 h due to the higher operating temperature, consequently acting towards higher hydrogen permeating flux. After 300 h at 420 °C, they reached average values of around 500 and 220, respectively, remaining constant up to 1100 h under operation. 

**Figure 3:**
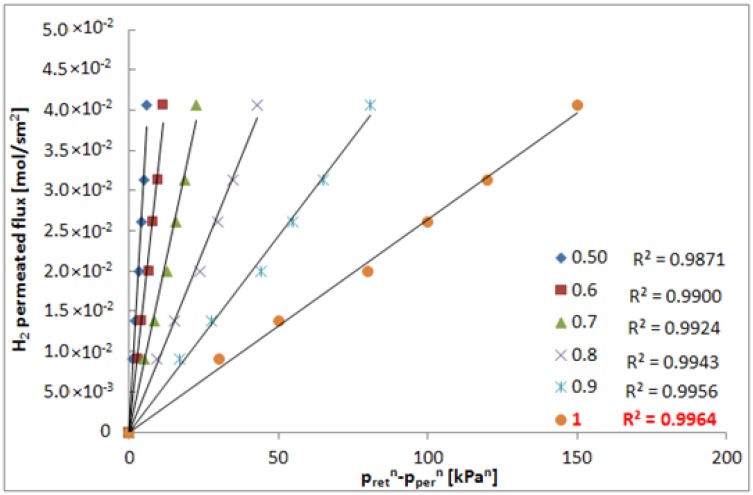
H_2_ permeating flux *vs*. the driving force at different “n” and T = 420 °C.

In the range 400–1000 h, when the membrane showed constant properties in terms of hydrogen permeating flux and ideal selectivities, in order to define the correct value of *n*, a series of hydrogen permeation tests was performed at different transmembrane pressures to calculate the linear regression factor (R^2^) at different “*n*”. As illustrated in Figure 3, the highest value of R^2^ was reached at *n* = 1, meaning that the permeation characteristics of the composite membrane are far from the Sieverts–Fick law [4], and, meanwhile, confirming a considerable presence of defects as pin-holes in the Pd-Au layer.

Furthermore, with the lifetime of the membrane around 1100 h under continuous operation, CH_4_ and CO_2_ permeation tests were also performed besides H_2_ and the results are summarized in Table 2. As it was mentioned earlier, hydrogen permeates through the Pd-Au layer by a solution-diffusion mechanism. On the contrary, the other gases permeate through the defects of the Pd-Au surface and the pores of the support with a Knudsen diffusion mechanism [36]. 

The H_2_/CO_2_ ideal selectivity of a bit less than 500 represents an interesting result for the separation of hydrogen and carbon dioxide mixtures. However, only a few results are available in the open literature about Pd-Au composite membranes applied in the field of gas separation and reaction testing, particularly evaluating the membrane aging. Then, a qualitative summary of the long-term characteristics of Pd-Au membranes from literature data are displayed in Table 3. Preparation method, thickness of the Pd-Au layer, operating conditions and H_2_ permeance as well as α_H2/He_ and reference are reported. As shown, the ideal selectivity α_H2/He_ of this work is the lowest compared to the other reported data, but it was calculated after almost 1000 h under operation, while the other data refer to the onset of the experimental tests or for a shorter range time under continuous operation. 

### 2.2. Methane Steam Reforming Reaction in the MR Housing the Pd-Au/PSS 

With the aim of examining the performance of the Pd-Au/PSS membrane in an MR, the MSR reaction was carried out (as reported by the square indicator in Figure 1) at 420 °C and 300 kPa of reaction pressure by using also a sweep-gas in the permeate stream in counter-current mode with respect to the feed. 

The endothermic reaction of MSR reaction can be expressed according to Equation (1):

CH_4_ + H_2_O = CO + 3H_2_, ΔH = 206 kJ/mol (1)

In the meanwhile, the water-gas shift (WGS) reaction takes place according to Equation (2):

CO + H_2_O = CO_2_ + H_2_, ΔH = −41.15 kJ/mol (2)

**Figure 4:**
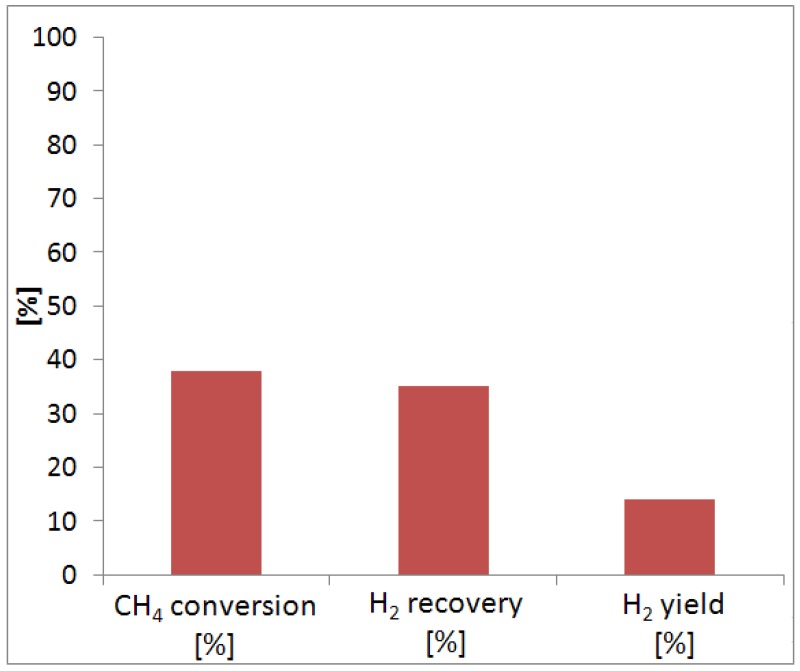
Methane conversion at 420 °C, 300 kPa and GHSV = 4100 h^−1^ during MSR reaction in the Pd-Au MR.

Table 4 resumes the operating conditions of MSR reaction, and Figure 4 sketches the reaction results in terms of methane conversion, hydrogen recovery and yield. 

The first reaction test was carried out at 420 °C, GHSV = 4100 h^−1^ and after 500 h of the membrane under pure gas permeation tests and with a constant hydrogen permeance. At these conditions, methane conversion is equal to 40% and the hydrogen recovery around 35%, while the yield is around 15%. The low conversion of methane is probably due to the low reaction temperature used during the tests. 

**Figure 5:**
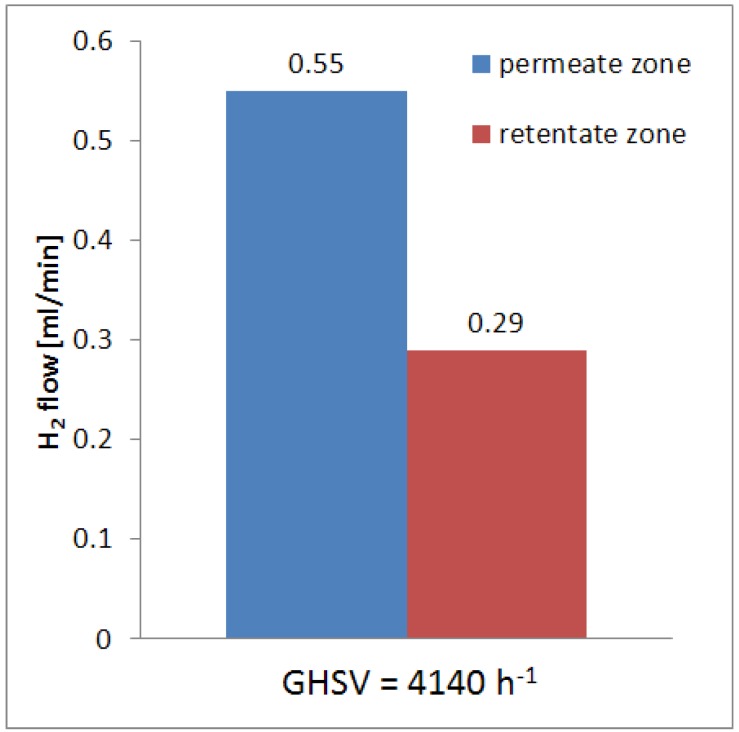
Hydrogen in the permeate and retentate streams during MSR reaction in the Pd-Au MR at 420 °C, 300 kPa and GHSV = 4100 h^−1^.

Furthermore, owing to a low value of hydrogen perm-selectivity the effect of the conversion shifting is not so much effective compared to dense Pd-based membranes, full hydrogen perm-selective. As a consequence, the hydrogen yield is also low as well as the hydrogen recovered in the permeated stream. Furthermore, as shown in Figure 5, the total hydrogen produced during the reaction was 0.84 mL/min, subdivided in the hydrogen in the permeate and retentate streams. 

All the results reported in Figure 4 and Figure 5 show a maximum error bar lower than 2%. 

Hence, in order to improve the methane conversion, the space velocity was decreased to favor higher residence time of the reactants in the reaction zone with a consequent longer contact time between the gas mixture and the catalyst. Nevertheless, at GHSV = 1100 h^−1^ and 420 °C, the hydrogen recovery increased up to 65%, but the conversion dropped to around 10%. This reverse phenomena can be a sequence of catalyst deactivation due to coke formation. 

As a consequence, the experimental tests at 1100 h^−1^ were repeated, and, once again, coke formation was verified. Then, after the reaction test, pure hydrogen was flowed into the reaction side (~3.0 × 10^−3^ mol/min) at 420 °C and for around 2 h. 

**Figure 6:**
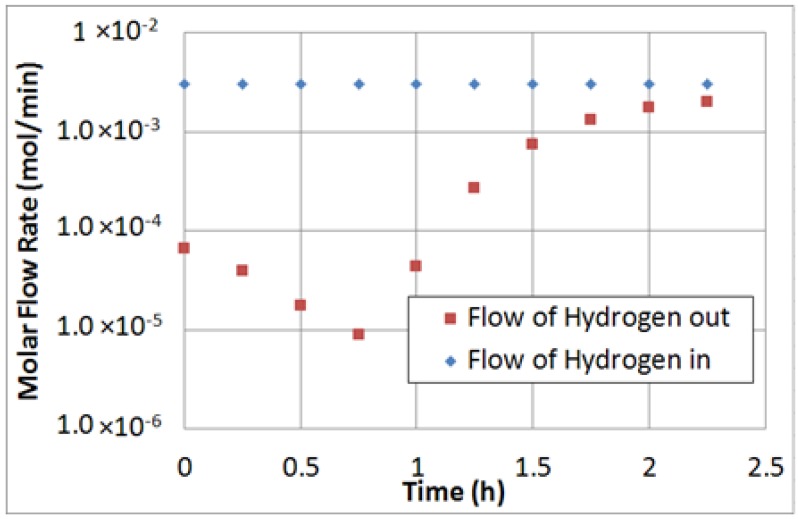
Molar flow rate of H_2_ in and out of the MR during coke deposition analysis.

**Figure 7:**
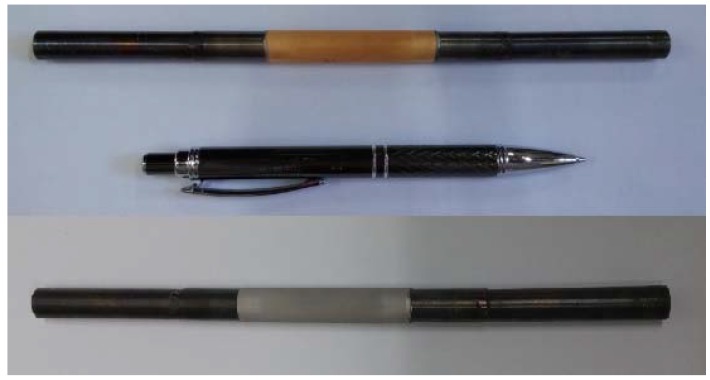
Picture of the supported Pd-Au membrane before tests (**top**) and after (**bottom**).

Figure 6 shows the feed and retentate molar flow rates of hydrogen. The results show that, particularly, in the first hour of operation, a substantial part of hydrogen fed to the MR was consumed in the catalytic bed, forming methane and indirectly confirming the coke deposition during the experimental tests. Indeed, during the hydrogen feeding procedure, methane was detected by analyzing the retentate stream to the GC. Hence, after almost 1 h under operation, the flow rate of methane formed during this procedure decreased gradually till 2.25 h, in which the inlet hydrogen stream was equal to the outlet stream. Successively, the reaction test was repeated, but the conversion remained still low and, then, the reaction tests were stopped. To verify the hydrogen permeation characteristic of the Pd-Au/PSS membrane after the reaction tests, permeation tests with pure H_2_, N_2_ and He were further performed. Unfortunately, both α_H2/N2_ and α_H2/He_ ideal selectivities dropped dramatically and, consequently, the MR was cooled down at ambient temperature, removing the membrane from the MR module. Figure 7 shows the status of the membrane before and after the experimental campaign, highlighting the color difference of the membrane surface, moved from the initial gold to gray. 

Gade *et al.* [37] supposed that the presence of H_2_S was responsible of the grain boundary attack with consequent loss of gold. However, the aforementioned authors stated that, in their opinion, the H_2_S attack was not the sole mechanism as a cause of gold depletion as in the case of this work. Therefore, in a next study, we will investigate in details what could be the mechanism for the change in color of the membrane surface from gold to gray during such a reaction as MSR in absence of H_2_S in the feed. 

## 3. Materials and Methods

### 3.1. Membrane Preparation 

Different techniques can be adopted for depositing palladium or its alloys onto porous substrates such as magnetron sputtering [38], spray pyrolysis [39], chemical vapor deposition (CVD) [40], physical vapor deposition [39,41] and electroless plating deposition (ELP) [42,43]. In this study, a dense Pd-Au layer (~7 μm thick) was deposited onto a PSS tubular support via the ELP method. 

The support was supplied by Pall AccuSep (New York, NY, USA) having 1.0 cm O.D. (AISI 316L porous tube) with an active length around 4 cm. The porous support was welded to two stainless steel tubes, and one of them is closed for facilitating the membrane housing in the MR module. Then, the total length of the membrane tube was 20 cm. The support was oxidized for 12 h at 500 °C. It was then graded with preactivated Al_2_O_3_ particles and cemented with Pd in order to decrease the surface pore size and narrow the surface pore size distribution and create the intermediate layer for avoiding intermetallic diffusion. 

**Figure 8:**
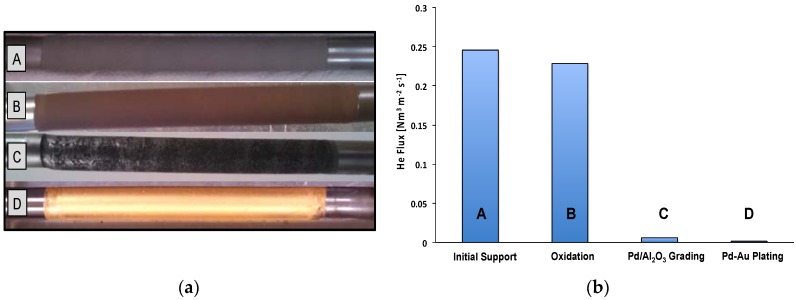
(**a**) fabrication Steps: [A] Initial support [B] Oxidation at 500 °C [C] Grading [D] Final Pd-Au metallic layer; (**b**) He permeating flux during membrane progress (Δp = 1 bar, T = 25 °C).

The surface activation was then carried out using the standard SnCl_2_-PdCl_2_ activation procedure and a thin layer of pure Pd was deposited by electroless plating technique. Successively, the Au deposition was performed by the method described in detail in Chen and Ma [29]. After each step of membrane preparation, the He permeance at room temperature was also measured (Figure 8a,b).

Hence, the Pd-Au/PSS membrane was allocated in the module and fixed by means of graphite gaskets and 3.0 g of Ni/Al_2_O_3_ commercial catalyst was packed in the annulus of the MR. To prevent the movement of catalyst particles, glass spheres were placed at each side of the catalytic bed. 

The MR was flowed with pure N_2_ at ambient temperature to check the presence of leakages. A thermocouple was used to measure the temperature of the MR module, which was heated up by using two electrical tapes up to achieving the required temperatures (300–420 °C). The retentate pressure was adjusted by utilizing a back-pressure regulator placed at the outlet side of this stream, while the permeate pressure was always kept at 100 kPa.

**Figure 9:**
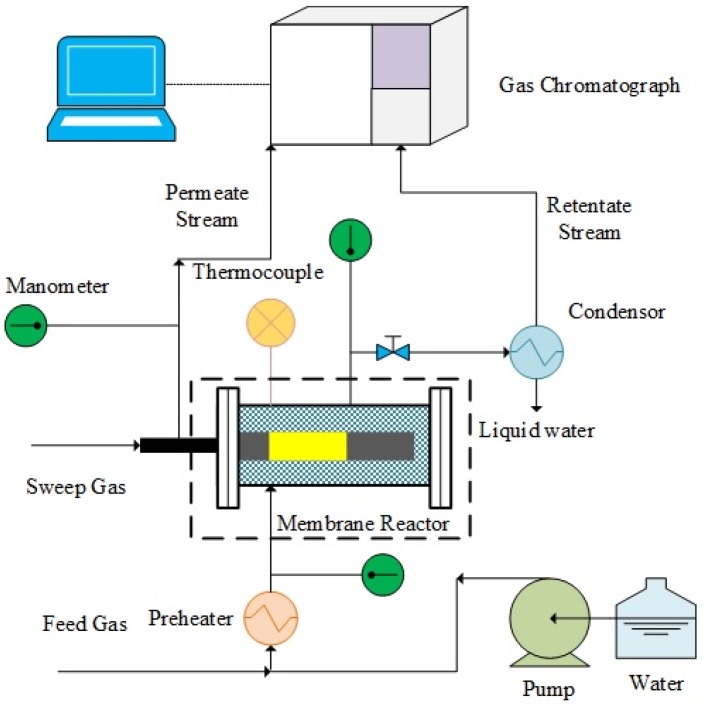
Schematic view of the experimental setup used for permeation and MSR reaction tests.

A scheme of the experimental setup used in this work is illustrated in Figure 9. In the case of permeation tests, pure gases (H_2_, N_2_, He, CH_4_ or CO_2_) were flowed into the MR module and the permeating flow rate was measured by means of bubble flow meters.

During the reaction tests of MSR, methane was mixed with deionized water (steam/methane = 3.5/1), which was pumped by a P680 HPLC pump (Dreieich, Germany), in a preheated chamber and, then, injected to the reaction zone. N_2_ was also used as an internal standard gas and as a sweep gas with a flow rate of ~25 mL/min, flowed in counter-current mode with respect to the feed. To remove the unreacted water from the retentate and, eventually, from the permeate streams, they were passed through a condenser. Then, the retentate and permeate dry streams were analyzed by means of a temperature programmed HP 6890 gas chromatograph (GC) (Foster City, CA, USA). Each experimental point reported in this work was constituted of, at least, ten reaction cycles at the same operating conditions in order to ensure the reproducibility of the results.

### 3.2. Permeation Tests 

Pure gases such as H_2_, N_2_, He, CH_4_ or CO_2_ were flowed into the membrane reactor module before reaction tests for studying the permeation characteristics of the supported Pd-Au membrane.

The pure gases were provided by cylinders. 

To analyze the hydrogen permeation characteristics of the Pd-Au/PSS membrane, Equation (3) was used as:

J_H2_ = P_H2_ (p^n^_H2,retentate_ − p^n^_H2,permeate_) (3)

where *J_H2_* is the hydrogen flux permeating through the membrane, *P_H2_* the hydrogen permeance, *p_H2,reaction_* and *p_H2,permeate_* the hydrogen partial pressures in the reaction and permeate sides, respectively. *n* is variable in the range 0.5–1 depending on the rate limiting step of hydrogen diffusion. More details about the deviation of Sieverts–Fick law (*n* = 0.5) were explained elsewhere [44].

The hydrogen over other pure gas ideal selectivity (α_H2/i_) was expressed as the ratio of the H_2_ permeating flux over the permeating flux of another pure gas of interest at the same transmembrane pressure, as reported in Equation (4):

α_H2/i_ = J_H2_/J_i_, i = (He, N_2_, CO_2_, CH_4_) (4)

Methane conversion was described by using Equation (5) during MSR reaction:

Conversion (%) = [(CO_out_ + CO_2out_)/CH_4 in_] × 100 (5)

where CO_out_ and CO_2out_ represent the outlet molar flow rates of CO and CO_2_, while CH_4,in_ represents the molar flow rate of methane in the reaction side.

Hydrogen recovery was defined by Equation (6):

Hydrogen Recovery (%) = [H_2-perm_/( H_2-perm_ + H_2-ret_)] × 100 (6)

where H_2-perm_ and H_2-ret_ are the molar flow rates of hydrogen in the permeate and retentate streams, respectively.

## 4. Conclusions

We investigated the performance of a supported Pd-Au membrane in terms of hydrogen permeance and ideal selectivity in pure gas permeation tests under long time continuous operation. The hydrogen permeance increased till achieving steady state conditions after around 400 h. Hence, at 50 kPa of transmembrane pressure and 420 °C, the composite membrane reached H_2_/N_2_ ideal selectivity of around 500 with an hydrogen permeance of 2.3 × 10^−2^ mol/m^2^·s, remaining stable up to 1100 h under operation. 

During MSR reaction tests, 40% of methane conversion and 35% of hydrogen recovery were reached in the MR. However, coke was formed during the reaction tests, and it was probably responsible of the irreversible loss of hydrogen permeation characteristics of the membrane. The MR was cooled at ambient temperature, and we observed that the color of the membrane surface moved from gold to gray. In the future, we will better investigate this effect, making more stable the alloy during the reaction tests, meanwhile, improving the hydrogen permeation selectivity. 

## Figures and Tables

**Table 1:** H_2_ and N_2_ permeating flux at 350 °C with the Pd-Au/PSS membrane at the onset of the experimental testing.

Pure gas (i)	Transmembrane Pressure (kPa)	Permeating Flux (mol/m^2^·s)	α_H2/i_
H_2_	50	3.83 × 10^−3^	1
	100	8.81 × 10^−3^	
N_2_	50	4.83 × 10^−5^	80
	100	9.13 × 10^−5^	~100

**Table 2:** Permeation test results after 1100 h of composite membrane under continuous operation at 420 °C.

Pure Gas-i	Molecular Weight (g/mol)	Transmembrane Pressure (kPa)	Permeating Flux (mol/m^2^·s)	α_H2/i_
H_2_	2.00	50	2.3 × 10^−2^	1
CH_4_	16.04	50	8.6 × 10^−5^	270
CO_2_	44.01	50	4.7 × 10^−5^	490

**Table 3:** A comparison between the long term characteristics of different Pd-Au membranes

Preparation Method	Pd-Au Layer (µm)	T (°C)	Δp (kPa)	H_2_ Permeance (Nm^3^ m^−2^ h^−1^ bar^−0.5^)	α_H2/He_	Ref.
Electroless Plating	Pd:6.1 Au:0.5	450	100	14.0 ± 2.4 (after 473 h in syngas atmosphere, 1200 kPa)	>160 000(initial)	[34]
Electroless Deposition	Pd:12.6Au:1.2	350	100	11.2 (after 150 h in pure H_2_ atmosphere)	900 (after 150 h)	[32]
Electroless Plating	Pd:14Au:0.9	450	100	5.5 (after 250 h in H_2_/N_2_ mixtures and syngas atmosphere, 1200 kPa)	>2700(Initial)	[35]
Electroless Deposition	Pd:8.2Au:0.16	450	100	8.3 ± 2.6 (after 250 h in H_2_/N_2_ mixture and syngas atmosphere 1200 kPa)	>1563(Initial)	[33]
Electroless Deposition	Pd-Au:7	420	50	5.6 * after 1000 h operation	220 (after 1000 h under operation)	This work

* The unit of this value is (Nm^3^ m^−2^ h^−1^ bar^−1^).

**Table 4:** Operating conditions during MSR reaction tests in the Pd-Au MR.

T = 420 °C	
p (retentate) = 300 kPa	
p (permeate) =100 kPa	
Steam/Methane = 3.5/1	
Sweep flow (N_2_) = 25 mL/min	
GHSV * = 4100 h^−1^	CH_4_ = 1.9 × 10^−3^ mol/min
	H_2_O = 6.7 ×10^−3^ mol/min

* Gas Hourly Space Velocity.

## Nomenclature

**Symbol**	**Definition**	**Unit**
B	Permeability	mol·m^−1^·s^−1^·kPa^−n^
dp	Pore Diameter	m
D_ki_	Knudsen Diffusivity	m^2^·s^−2^
J	Permeation Flux	mol·m^−2^·s^−1^
M	Molecular Weight	kg/mol
p	Partial Pressure	kPa
P	Total Pressure	kPa
t	Time	h
T	Temperature	°C

## Greek Letters

**Symbol**	**Definition**	**Unit**
α	Ideal Selectivity	
δ	Membrane Thickness	m

## Subscripts

**Symbol**	**Definition**	**Unit**
i	Species	

## Abbreviations

GC	Gas Chromatograph	
GHSV	Gas Hourly Space Velocity	h^−1^
MR	Membrane Reactor	
MSR	Methane Steam Reforming	
PSS	Porous Stainless Steel	
